# Amphipathic Janus Membrane with Hierarchical Multiscale Hyperporous Structure for Interfacial Catalysis

**DOI:** 10.3390/membranes10080162

**Published:** 2020-07-23

**Authors:** Yakai Lin, Yuanyuan Liu, Yicheng Su, Lin Wang, Yuanhui Tang, Tianyin Liu, Liwei Ren, Xiaolin Wang

**Affiliations:** 1 Beijing Key Laboratory of Membrane Materials and Engineering, Department of Chemical Engineering, Tsinghua University, Beijing 100084, China; yk_lin@tsinghua.edu.cn (Y.L.); suyc@casim.cn (Y.S.); wanglin891208@mail.tsinghua.edu.cn (L.W.); liutianyin@genertec.com.cn (T.L.); 2 Aerospace Institute of Advanced Materials & Processing Technology, China Aerospace Science & Industry Corp., Beijing 100084, China; liuyy07@163.com; 3 College of Chemistry and Environmental Engineering, China University of Mining and Technology, Beijing 100083, China; tangyuanhui@126.com; 4 College of Biological and Pharmaceutical Sciences, China Three Gorges University, Yichang 443002, China; renliwei@ctgu.edu.cn

**Keywords:** janus membrane, amphipathic, multiscale, interfacial catalysis

## Abstract

The rational design and realization of multiscale porous structures has been a long-standing challenge in membrane science. Block copolymers (BCPs) with their self-assembly-enabled nanodomains have the potential to make structural breakthroughs. An amphipathic Janus membrane, with a hierarchical multiscale hyperporous structure constituted by polystyrene-b-poly(4-vinylpyridine) (PS4VP) and polyvinylidene fluoride (PVDF) blocks, was designed and synthesized in this work. Hydrophobic PVDF dominated one side of the membrane, and hydrophilic PS4VP, with nanopores that formed inside the macroporous channels of PVDF via a self-assembly approach, dominated the other side. Candida Rugosa Lipase (CRL), as a model biocatalyst, was immobilized in the PS4VP nanopores via injection. The immobilized lipase was exactly suspended at the interface of the organic and aqueous phases, owing to the amphipathic property of the Janus membrane. The designed structures and catalysis performances were further characterized. The immobilized lipase exhibited a three times higher specific activity than free lipase, and the relative activity still remained above 90% after 10 cycles of reusing, indicating the observable promotion and the guaranteed stability of the Janus membrane in interfacial catalysis. This work provided a general, facile and unique example for the design and synthesis of a hierarchical multiscale hyperporous membrane for interfacial catalysis.

## 1. Introduction

The scalable and sustainable manufacture of hyperporous films, with precisely regulated molecular transport, have wide applications in electrochemistry, catalytic fields and sensors [1,2,3,4]. Strategies for facilitating molecular transport involve reducing the film thickness down to the nanoscale or introducing film porosity by designing nanoarchitectures [1,5]. The rapid development of two-dimensional materials, such as graphene and porous nanosheets, have paved the way for the formation of the ultra-thin and porous thin films [6,7,8,9]. The bottom-up method for building transport barriers has the demonstrated advantages of ultra-thin thickness and tailored pore sizes [10,11]. Alternatively, block copolymers (BCPs) promise the scalable, feasible fabrication of isoporous structures with nanopores via self-assembly [12,13,14,15]. In many cases, well-defined surface isoporosity can be successfully obtained via various methods. However, most methods can hardly lead to tortuous channels beneath the porous layer, making it challenging to maintain the vertical alignment of pores in thicker films. Thus, the obtained membrane usually exhibits an inferior selectivity compared to the state-of-the-art ultra-thin films, due to the limited molecular transport [16].

A shift to hyperporous BCP membranes, with hierarchical multiscale pore structures, enables an economical approach [1]. Isoporous nanochannels with short pathways are suitable for precisely tailored two-way molecular transport, while the macroporous frameworks maintain integrity and mechanical robustness, which are essential prerequisite for separator membranes [14,17]. The typical methods for developing hyperporous BCP membranes include self-assembly and post-synthesis treatments [18,19]. However, these approaches include a complex templating process and use BCP as a substrate, leading to high manufacturing cost, and a lack of tunability and generality tailored to its use [1,20]. Using heterogeneous materials enables an economical and versatile approach. Here, we propose a new strategy for enabling a hierarchical structure with a "locked", highly-ordered nanopores layer in the macroporous backbone, by combining a dual-compositional self-assembly and a solvent-tailoring method.

The hyperporous membranes are ideal candidates as immobilization supports in biocatalysts due to their well-ordered and symmetric nanopores [4,21]. In particular, interfacial catalysis, which occurs at the interface of two immiscible liquids, is essential for highly biocompatible applications, including biopharmaceuticals, food processing and ester hydrolysis [22,23,24]. Interfacial catalysis is known for its slow kinetics and complexity, and the exact distribution of catalysts at the interface is of great importance [25,26]. Recent advances in the tailoring of interfacial location and distribution, and the affinity of the nanomaterial catalysts, enable fast and efficient interfacial catalysis [20,24,27]. However, various methods have been applied to preparing biocatalytic membranes, such as a layer-by-layer (LbL) self-assembly method, and surface covalent modification [28,29]. Nevertheless, we hypothesize that the enzyme-loaded membranes with amphipathic affinity can lead to efficient catalysis location and product separation. Furthermore, the highly porous and defect-free films improve the substrate transport and the acquired reusability of the biocatalyst.

**Figure 1:**
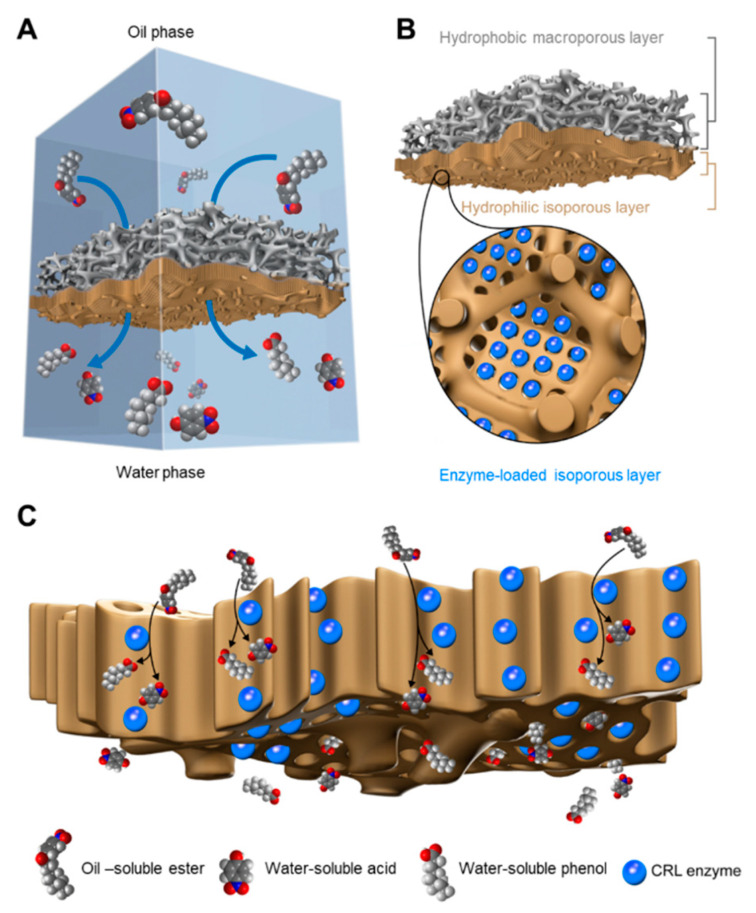
Design scheme of the interfacial catalysis of lipase loaded in a Janus membrane. (**A**) Schematic process of a interfacial catalysis; (**B**) Illustration of the hierarchical multiscale hyperporous structure of lipase loaded Janus membrane; (**C**) Schematic catalysis process inside the hyperporous structure.

Here, a Janus membrane with a hierarchical multiscale hyperporous structure is designed for directional transport and amphipathic affinity. Meanwhile, the hyperporous structure with chemical functionality enables facile bio-functionalization via a loading enzyme (Figure 1). In general, polyvinylidene fluoride(PVDF) blocks provide stable and hydrophobic frameworks, and the bi-continuous macroporosity (pore size c.a. > 1 µm) of PVDF provides space for the formation of finer hierarchical structures. The hydrophobic PVDF pore wall is compatible with the isoporous layer via the forming of hydrophobic interactions between the hydrophobic polystyrene (PS) nanodomains of poly(4-vinylpyridine) (PS4VP) chains, while the other end of the chain possesses functionalizedP4VP groups that are exposed to the inner surface of the well-organized isoporoes [14,30]. Thus, PS4VP forms a defect-free hierarchical multiscale structure, with well-ordered mesopores in each macrovoid of the PVDF, via self-assembly. These PS4VP isopores, with penetrable porosity and chemically functionalized pore walls, enable the facile immobilization of nanoscale enzymes, such as Candida Rugosa Lipase (CRL) [23,31,32]. Theoretically, the lipase with the catalytic function of hydrolyzing ester bonds is exceptionally fit for the role of the hydrophobic substrate and the hydrophilic product at a heterogeneous interface. Through this approach, the active site of the lipase that is immobilized in the highly porous structure is exposed to small molecular substrates, and enables the rapid release of products. Hence, the amphipathic property of the Janus membrane facilitates the optimum and stable contact of the substrates/products at the interface, forming a catalytically active membrane with low transport resistance for heterogeneous reactions at the interface [33,34,35,36].

## 2. Materials and Methods

### 2.1. Materials 

DMF, 1,4-dioxane (DOX), ACE, DMSO, polyethylene oxide (PEO, weight average molecular weight (Mw) = 2,000, 6,000, 20,000, 100,000, 200,000, 300,000 and 600,000), Victiria blue, HAc and NaAc were supplied by Sinopharm Chemical Reage nt Beijing Co., Ltd., Bejing, China. Candida rugosa lipase (CRL, type Ⅶ, ≥700 unit/mg) with a molecular weight of 58 kDa [37] was obtained from Sigma-Aldrich LLC., St. Louis, US. 4-nitrophenyl octanoate and 4-nitrophenol were purchased from Alfa Aesar LLC., Heysham, UK). PS4VP samples (number P10900-S4VP, number average molecular weight (Mn) = 190,000-b-64,000; Mw/Mn = 1.14) were purchased from Polymer Source, Inc., Dorval, Canada. PVDF commercial flat sheet membranes (0.22-µm pore size, hydrophobic) were purchased from EMD Millipore Co., Darmstadt, Germany.

### 2.2. Preparation of PS4VP/PVDF Janus Membrane 

PS4VP powder was dispersed in a dope solution consisting of DMF, DOX and ACE with a weight ratio of 12:21:8. The solution was stirred for 24 h at 25 °C and stored for another 24 h to remove bubbles. The PVDF flat sheet membrane was immersed in DOX for 5 s, and then dried softly with filter paper. The casting solution was doped on the surface of the PVDF membrane and the excess solution was removed with a rubber roller. After 5 s of evaporation, the membrane was immersed in a water bath. Finally, the PS4VP/PVDF Janus membrane was dried under a vacuum at 40 °C overnight.

### 2.3. Loading Process of CRL into Janus Membrane

Enzyme solution of 1 g/L was prepared by dissolving CRL in buffer solution of HAc/NaAc (20 mmol/L, pH = 5.0, 4 °C). The solution was centrifuged at 6000 rpm for 5 min and the supernatant was obtained for the loading processes. Prior to CRL loading, the CRL supernatants were diluted to 0.05, 0.075, 0.1, 0.2 and 0.3 g/L according to the initial concentration. Then, the PS4VP/PVDF membrane was put in a filter together with non-woven fabric supporting the PS4VP side. A total of 0.5 mL of diluted CRL solution was added in and pushed through the syringe. Finally, the membrane loaded with CRL was taken from the filter and washed with DI water.

The adsorption efficiency (*E*_a_) of CRL in the loading process can be obtained by:

(1) $$E_{a}=(1-\frac{A_{e}}{A_{i}})*100\%$$

*A*_e_ and *A*_i_ are the UV absorbance of the effusive and initial CRL solutions at 280 nm, respectively. The amount of CRL loaded in the Janus membrane can be calculated by:

m = *E*_a_∙*V*_0_∙*C*_o_ (2)

*V*_0_ and *C*_o_ are the initial volume and concentration of the CRL solution, respectively. 

Fluorescent labeling process of CRL: Fluorescein isothiocyanate (FTIC) was used for CRL labeling in the Janus membrane. A Na2CO3/NaHCO3 buffer solution with a concentration of 50 mmol and pH = 9.41 was prepared. CRL was added in and dissolved to obtain an 8 g/L solution. Then it was centrifuged, and the supernatant was obtained for labeling. FTIC of 12.6 mg was added into 630 µL DMSO and stirred until homogeneous. The FTIC solution was added into the CRL solution for reaction in the dark for 12 h. Then, the solution was sealed in a dialysis bag. The dialysis was performed in a HAc/NaAC buffer solution (20 mmol/L, pH = 5.0) for 12 d (The buffer solution was replaced every 24 h). Finally, the labeled CRL solution was diluted to 1 g/L for use.

Rejection tests: The rejection of the membranes was measured using different molecular weights of PEO via a dead-end permeation cell with an effective membrane area of 4.6 cm^2^. First, PEO feed solutions with a concentration of approximately 1000 ppm were prepared. Then, the tests were performed at 25 ± 0.5 °C with a pressure of 0.3 MPa. The concentrations of the feed and permeate solutions were precisely determined by a total organic carbon analyzer (TOC, TOC-Vcpn, Shimadzu, Kyoto, Japan). The molecular weight cut-off (MWCO) was defined as the molecular weight of the molecule that was 90% retained by the membrane.

Diffusion test: The diffusion test was applied in a home-made experiment and the membranes were carefully sealed in the experiment by copper tape. In a typical diffusion test, one compartment (referred to as the permeate) is filled with DI water to fully wet the membrane. Then, the feed compartment is filled with Victoria blue solution. During the test, small amounts of the solution in the permeate compartment are taken out for measuring the concentrations of Victoria blue. The concentration of Victoria blue was also determined by the TOC analyzer. 

### 2.4. Catalysis Tests

The hydrolysis of 4-nitrophenyl octanoate was used as a model reaction for interfacial catalysis. A 10 × 10 × 43-mm quartz vessel was used as the reaction container. First, 3 mL of HAc/NaAc buffer solution (pH 5.0) was added into the vessel. Then the catalyst, CRL@PS4VP/PVDF, was cut into a 9-mm-diameter piece and carefully put onto the solution. Note that the PS4VP-filled PVDF side faced the water solution. Then, a certain amount of hexane containing 4-nitrophenyl octanoate was carefully added into the vessel. On account of the amphipathic property of the Janus membrane, CRL@PS4VP/PVDF will float exactly at the interface. Next, the vessel was sealed and put into a 40 °C oven for reaction. At certain time intervals it was taken out for testing with UV-vis spectrometer (UV-vis, TU-1810, Persee General Instrument, Beijing, China), under 348 nm directly. The yield curve of 4-nitrophenol can be calculated as below:

(3) $$y=\frac{C_{t}}{C_{end}}\times 100\%$$

in which y stands for the yield curve of 4-nitrophenol, $C_{t}$ and $C_{\mathrm{end}}$ stand for the concentrations of produced 4-nitrophenol in water at time t and the end of the reaction, respectively. 

The specific activity of the immobilized or free lipase can be calculated through the molar quantity of the produced 4-nitrophenol in the first 10 min, according to the yield curve of 4-nitrophenol.

### 2.5. Other Characteristics

The morphologies of the membranes were obtained by a field emission scanning electron microscope (FESEM, 6301F, JEOL Ltd., Akishima, Japan) at an accelerating voltage of 3 kV. In the meantime, energy dispersive X ray spectroscopy was used to acquire the surface compositions in detail. Chemical composition was characterized using attenuated total reflectance–Fourier transform infrared spectroscopy (ATR-FTIR, Nicolet 6700, Thermo Fisher Scientific Inc., Waltham, MA, USA), and the cross-sectional compositions were measured through the mapping technique of ATR-FTIR spectroscopy with an infrared microscope (Continuμm, Thermo Fisher Scientific Inc., Waltham, MA, USA). The mechanical properties of membranes were measured by a material test machine (AGS-J20N, Shimadzu Co., Ltd., Kyoto, Japan) at a loading velocity of 10 mm/min. A laser scanning confocal microscope (LSCM, LSM780, Zeiss, Oberkochen, Germany) was used to detect the labeling enzyme in the membrane. A nitrogen adsorption desorption test was performed to calculate the pore size distribution curve, from 2 to 100 nm (ASAP 2020, Micromeritics, Norcross, GA, USA).

## 3. Results and Discussion

**Figure 2:**
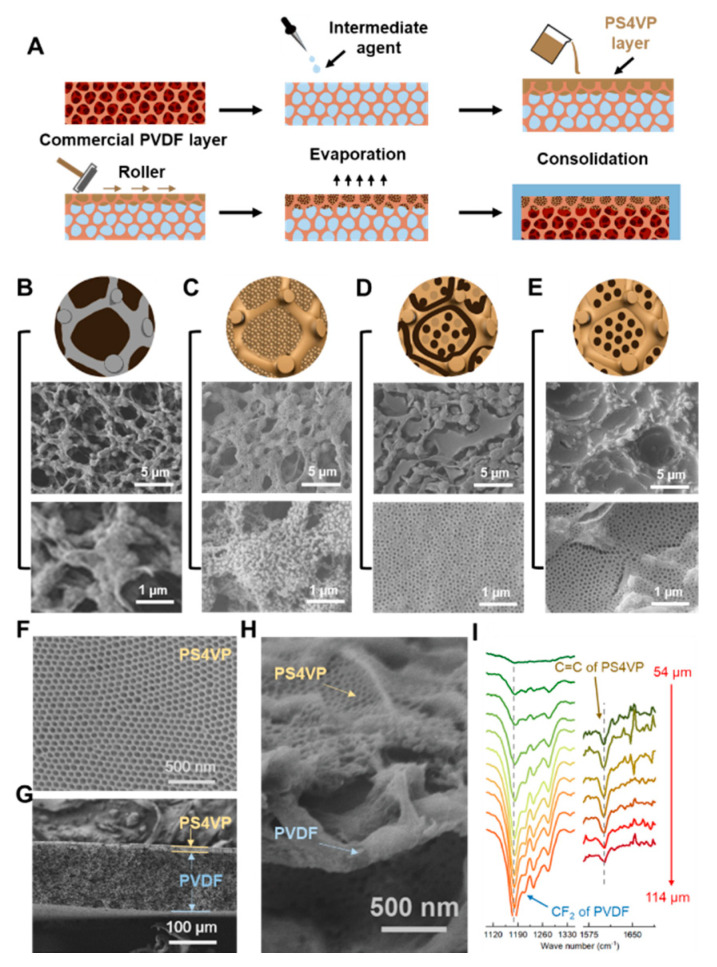
Fabrication and characterization of the PS4VP/PVDF Janus membrane. (**A**) Fabrication of the Janus membrane by SNIPS method. (**B**–**E**) FESEM images of PS4VP/PVDF membranes employing different ITAs. (**B**): pure PVDF and no ITA, (**C**): DMSO, (**D**): TEP, (**E**): DOX. (**F**) FESEM image of pure PS4VP membrane. (**G**) Cross-sectional FESEM images of PS4VP/PVDF Janus membrane. (**H**) Enlarged cross-sectional FESEM images of the layer filled with PS4VP of the Janus membrane. (**I**) FT-IR spectra of the PS4VP- filled PVDF layer, which show that the thickness is about 10 µm and the length of the facula area is 50 µm.

The well-ordered PS4VP structure inside the PVDF macropores was achieved by a self-assembly and non-solvent-induced phase separation (SNIPS) method (Figure 2A). The whole process simply requires dip-coating, evaporation and consolidation steps. Evaporation before the full phase conversion creates a concentration gradient beneath the surface of BCP membranes, which leads to a self-assembly morphology formation at the top with a range of hundreds of nanometers. The typical SNIPS methods (Figure S1a) only forms a dual-layer membrane, with a thick PS4VP layer covering the PVDF layer [14]. In order to have an isoporous morphology immediately at the surface of the PVDF pores, a planning step with a roller was done before evaporation in our method. The finely tailored conditions warrant the formation of PS4VP inside the PVDF pores. Further, the intermediate treatment agents (ITAs) were employed before dip-coating to ensure well self-assembled behavior inside pores of PVDF. It adjusted the compatibility between two polymers, and controlled the penetrative thickness inside the PVDF pores. Commercial PVDF membranes have macropores over 1 µm (Figure 2B). After dip-coating the PS4VP with the SNIPS method, using different ITAs, huge differences occurred. Spheres, isoporous morphologies with cracks, and normal isoporous structures were formed when using DMSO (Figure 2C), TEP (Figure 2D) and DOX (Figure 2E) as ITA, respectively. Comparing the Hansen solubility parameters of DMSO (δ_t_ = 26.7), TEP (δ_t_ = 22.2) and DOX (δ_t_ = 20.5), the DOX is the most similar to the solvent in casting solution (δ_t_ = 20.9) (Table S1). Solvents with similar properties to the casting solution will achieve similar morphologies. Thus, while using DOX as ITA, not only was the well-ordered morphology formed inside the PVDF backbones, but the cracks between skeletons and pore domains were reduced as much as possible. Finally, the Janus membrane was formed with the PS4VP domains holding high-ordered morphologies (Figure 2F), and the layer was filled with a PS4VP of 10-µm thickness (Figure 2G). Here, the PS4VP isoporous membranes were formed individually with the support of the PVDF backbone frameworks, and the spaces between the large backbones were empty. This provides the low density and large porosity of the PS4VP-filled PVDF layer (Figure 2H). The loose framework of the Janus membrane literally has no resistance against the small molecules passing through these holes. The thickness of the PS4VP-filled PVDF layer was also confirmed by FTIR mapping. The characteristic peak of PVDF (CF2), strengthened at 1181 cm^−1^ as the peak of PS4VP (C=C), vanished at 1597 cm^−1^ when scanning from the surface to the bulk domain (Figure 2I). The thickness can be calculated by scanning length (60 µm) minus facula length (50 µm), and the results match with those observed from the SEM images. Furthermore, the surface FTIR and EDX tests of the layer filled with PS4VP thoroughly confirmed the hierarchical multiscale hyperporous structure of the membrane (Figures S2 and S3).

**Figure 3:**
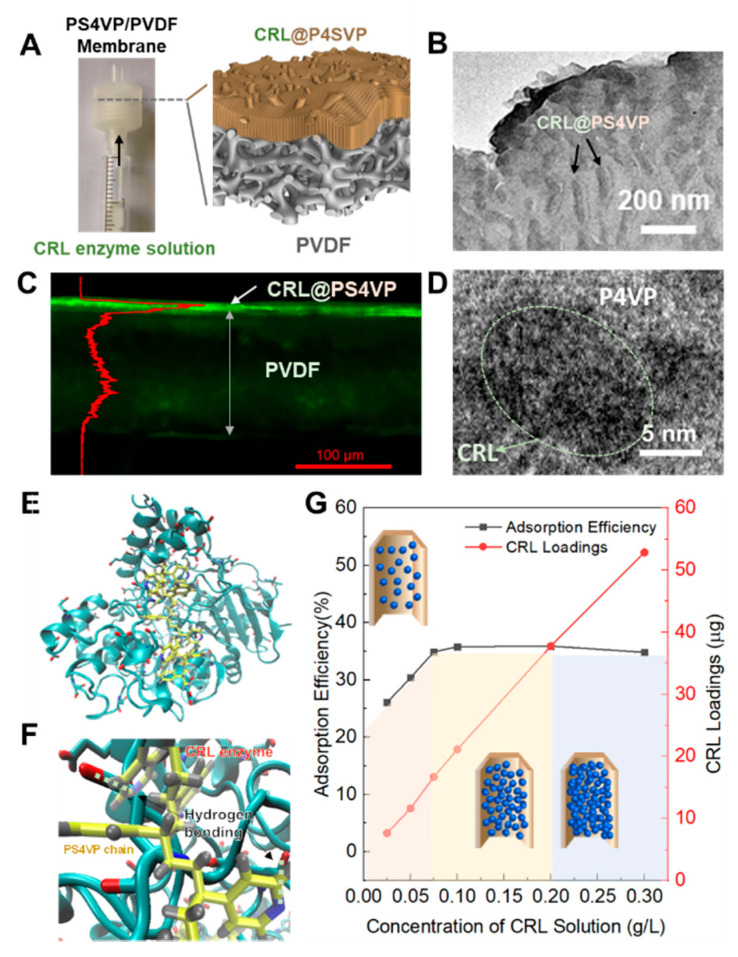
Loading CRL into the PS4VP/PVDF membrane. (**A**) Scheme of the loading process. (**B**) TEM images of CRL@PS4VP/PVDF. (**C**) EDX image of CRL@PS4VP/PVDF. The green shining part shows the existence of CRL. (**D**) Enlarged TEM images of the PS4VP area in CRL@PS4VP/PVDF (**B**–**D**): the initial concentration of CRL solution was 0.1 g/L). (**E**,**F**) Scheme of the interaction between CRL and PS4VP blocks (reseda: CRL, red: the glutamic and aspartic acids of CRL, yellow: PS4VP blocks, gray: the pyridines of PS4VP). (**G**) CRL adsorption efficiency and loading curves at different initial concentrations of CRL solutions.

CRL was then loaded into the Janus membrane just by an injector (Figure 3A and Figure S4). It is supposed that the size effects of CRL and the interaction with PS4VP both contributed to the CRL adsorption. As a single CRL molecule is about 3 × 4 × 5 nm, it is more consistent with the pore size of PS4VP than that of PVDF. Figure S5 shows that CRL was adsorbed in the pores with the range of 2–10 nm, leaving channels of about 40 nm for substrate and product transport through the PS4VP-filled PVDF layer. The TEM images of the PS4VP-filled PVDF layer after loading enzymes also confirm the existence of CRL. The bigger channels can be seen clearly in Figure 3B; also, the walls of the PS4VP-filled PVDF layer are rather thick compared with the no-enzyme-loaded ones (Figure S6a). In order to further confirm the location of CRL in the whole membrane, a fluorescent labelling method was employed. Once labeled by fluorescein isothiocyanate, CRL will glow green under a laser scanning confocal microscope. Figure 3C indicates that CRL was concentrated in the top range, about 10 µm, which matches with the thickness of the PS4VP-filled PVDF layer. The bulk PVDF part shows the minimal existence of CRLs, which proves that the pores of PVDF over 1 µm were too large to adsorb enzymes. Meanwhile, the existence of CRL surrounded by P4VP blocks was observed in the enlarged TEM images (Figure 3D). Except for the size effect, the interactions between the side chains of PS4VP and the external surface of the enzyme should also be considered. Some superficial amino acids of CRL, such as glutamic and aspartic acids, provide abundant free carboxyl groups (Figure 3E). There should at least be electrostatic interactions as well as hydrogen bonds between the carboxyl groups of the two electronegative amino acids and the pyridines of the PS4VP blocks (Figure 3F). The interactions could also be confirmed by adsorption efficiency versus CRL loading curves (Figure 3G). Here, the adsorption efficiency is defined as the concentration ratio of the CRL solution after and before injection. As the initial concentration of the CRL solution increased, the amount of loaded CRL rose linearly in the full range of testing. However, the adsorption efficiency stopped increasing when the initial concentration of the CRL solution reached 0.1 g/L. At the turning point, it is assumed that the loaded CRL in the pores of the PS4VP-filled PVDF layer reached a critical state, similar to the single layer-saturated adsorption. Furthermore, due to the funnel-like channels of the Janus membrane, there was still enough space for enzyme stacking, as in the 40-nm mesopores of the PS4VP-filled PVDF layer, even though the smaller pores of about 2–10 nm were filled with CRL.

**Figure 4:**
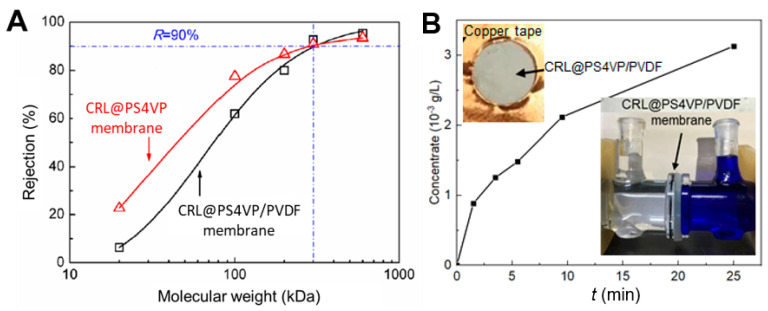
Penetration performance. (**A**) MWCO curves of the CRL@PS4VP/PVDF membrane and CRL@PS4VP membrane. (**B**) Diffusion curve of Victoria blue dye (Mw = 506.09 Da) through the CRL@PS4VP/PVDF membrane.

The designed hyperporous membrane with a hierarchical multiscale structure may be an ideal support for biomacromolecules, while also featuring little penetration resistance to other small molecules. The low tortuosity and abundance of space in the membranes may benefit the penetration performance, which is also very critical for interface catalysis, which usually consists of both diffusion and reaction steps. The performance of molecular transport through the Janus membrane was further verified by the diffusion and pressure-driven filtration of the enzyme analogue and small-molecule dye, respectively (Figure 4A,B). The rejections of PEG oligomers (molecular weight of 20–600 kDa) were tested to obtain theMWCO curves of the PS4VP/PVDF membrane, in contrast to a typical single layer PS4VP membrane (Figure 4A). Both the single layer PS4VP membrane and the PS4VP/PVDF membrane shared a same MWCO of ~300 kDa (29 nm). The Janus PS4VP/PVDF membrane exhibited a reduced rejection of PEG-20 kDa (<5%, 7.4 nm) and PEG-100 kDa (~60%, 16.5 nm) compared to those of the single layer PS4VP membrane (20% of PEG-20 kDa and 80% of PEG-100 kDa), highlighting the rapid transport through the hierarchical hyperpores, comprising an ultra-thin isoporous layer and macroporous backbones. The influence of enzyme loading on small molecule transport was verified by the U-cell diffusion of dye molecules (VB, Mw = 506 Da, Deff = 0.67 nm). The CRL-loaded Janus membrane, i.e. CRL@PS4VP/PVDF, demonstrated a VB diffusion rate (6.64 × 10^−2^ mmol L^−1^ h^−1^) close to the bulk Janus membrane. As expected, the low tortuosity and abundant in space of the Janus membrane, and even the CRL-loaded membrane, benefited the transport of small molecules, e.g., substrates and products. In addition, the integrality of the structural changes before and after CRL loading provided a basic guarantee of the penetration performance of CRL@PS4VP/PVDF (see TEM figures in Figure 3B and Figure S6a,b). 

**Figure 5:**
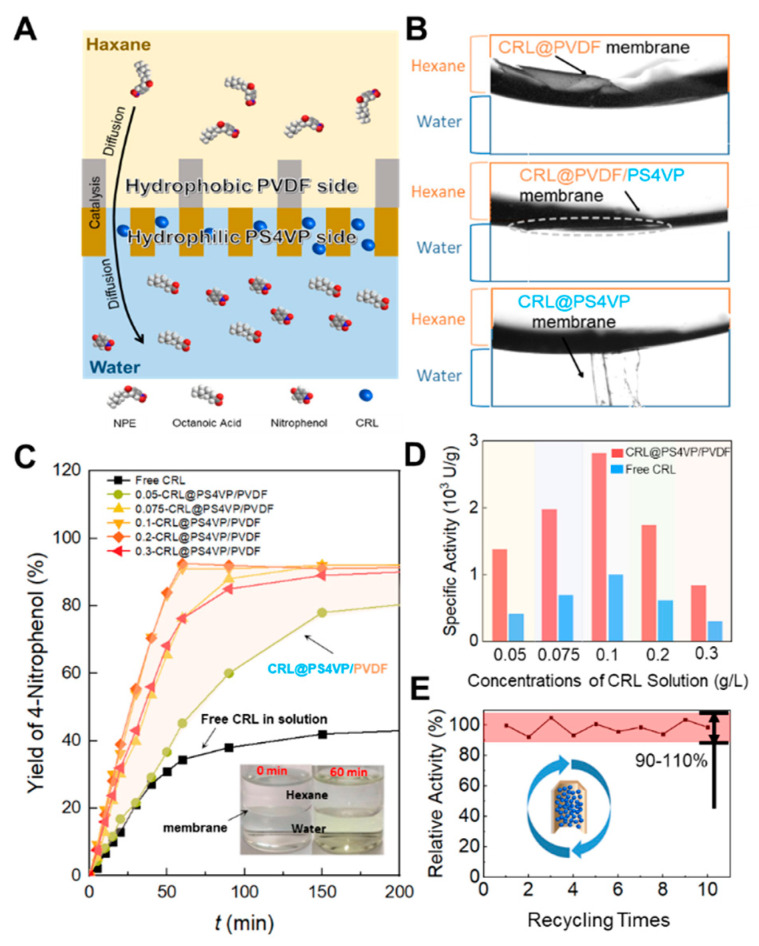
Interfacial catalysis performances of CRL@PS4VP/PVDF. (**A**) Scheme of the catalytic process at the interface of organic and aqueous phases. (**B**) Microscope images of the membranes floating at the interface. (**C**) Yield curves of 4-nitrophenol catalyzed by CRL@PS4VP/PVDFs and free CRL, note that the number before CRL@PS4VP/PVDF means the initial concentration of the CRL solution when CRL was loaded into the Janus membrane, and free CRL was add as a 0.1 g/L solution to aqueous phase with continuous and drastic stirring. (**D**) Specific activities of CRL@PS4VP/PVDFs and the same dose of free CRL. (**E**) Relative activities of CRL@PS4VP/PVDF during the 10 recycling times of catalysis.

For a typical heterogeneous interface reaction, the hydrophobic substrates should firstly diffuse from the organic phase to the interface, and be converted by catalysts into water-soluble products, then diffuse to the aqueous phase. We used a model reaction of the hydrolysis of 4-nitrophenyl octanoate, which diffused from hexane to the interface and contacted with CRL (loaded in CRL@PS4VP/PVDF) to produce octanoic acid and 4-nitrophenol. This was particularly the case for the water-soluble 4-nitrophenol transferred through the membrane into the aqueous phase, which made the bulky solution visible yellow (Figure 5A,C). By resorting to the amphipathic property of the Janus membrane, CRL@PS4VP/PVDF floated exactly at the interface, in which the bulk PVDF side faced the organic phase and the PS4VP-filled PVDF side faced the aqueous phase. CRL loaded in the PS4VP pores of the Janus membrane are thus near the hexane and water interface. As control, we prepared both pure PVDF and PS4VP membranes, and employed them to load CRL, namely CRL@PVDF and CRL@PS4VP, respectively. For CRL@PVDF, most of the membrane was exposed inside the organic phase. In contrast, some parts of CRL@PS4VP were prone to sink into the aqueous phase, and both of them gave inefficient catalytic performances (Figure 5B).

We further tailored the CRL loading in the Janus membrane. Figure 5C shows the effects of CRL loadings on the heterogeneous reaction. In general, the yield of 4-nitrophenol achieved a maximum of about 92% for some CRL@PS4VP/PVDFs within an hour, even though the mixing equipment was not required during the whole process. As time increased, all the CRL@PS4VP/PVDFs tended to reach the same yield, but with different speeds as load changes. However, free CRL produced a much lower yield (about 40%), in spite of the diffusion being facilitated by continuous and drastic stirring. In addition, increasing the dosage of free CRL did not offer the yield of 4-nitrophenol a significant improvement. This shows that the separation of substrates and products, which existed in the two sides of the Janus membrane respectively, should contribute to changing the equilibrium of the reaction and accumulating more products. For CRL@PS4VP/PVDF, the yield curves were nearly the same when employing CRL solutions of 0.2 g/L and 0.1 g/L, and they were much higher than that achieved using 0.3 g/L. Further characterization was performed by comparing the specific activities of CRL@PS4VP/PVDFs (Figure 5D). Firstly, each CRL@PS4VP/PVDF exhibited much greater activity than free CRL, under the precondition that the amount of CRL loaded in CRL@PS4VP/PVDF equaled that used as the free state. At the initial concentration of 0.1 g/L, as an example, the specific activity of the prepared CRL@PS4VP/PVDF was three times greater than the corresponding free CRL. Besides the separation effect of substrates and products, the coordinated pore size with CRL and the lower transport resistance of the Janus membrane contributed to the higher activity of CRL@PS4VP/PVDF. Next, CRL overloading in the Janus membrane limited the positive effect of increasing the specific activity of the prepared CRL@PS4VP/PVDF. When the initial concentration was above 0.1 g/L, the specific activities of CRL@PS4VP/PVDFs decreased dramatically. As the enzyme molecules accumulated inside the pores of the PS4VP-filled PVDF layer, the inner enzyme could not contact the substrates, and the active sites of the external enzyme might be allosteric, as caused by the congestion in the pores. This result also matched with the turning point of the adsorption efficiency of CRL in the Janus membrane (Figure 3G).

The optimal loading condition was obtained by employing the CRL solution of 0.1 g/L. Under this condition, the prepared CRL@PS4VP/PVDF could be well reused, and the relative activities mildly floated between 90% and 110% (setting the first time as 100%) during the 10 recycling durations of catalysis (Figure 5E). This indicated that CRL was loaded in the PS4VP pores of Janus membrane, and the generated interactions between the enzyme molecules and the P4VP blocks (mainly electrostatic interaction and hydrogen bonds, as noted above) were strong enough to prevent the CRL leaching to either the organic or the aqueous phase in the heterogeneous reaction system. On the other hand, the involvement of robust PVDF backbones provided the high mechanical strength of the Janus membrane and CRL@PS4VP/PVDF, making them suitable for long-term use (Figure S8 and Table S2).

## 4. Conclusions

In summary, we put forward a general, facile and unique example of the design and synthesis of a hierarchical multiscale hyperporous membrane (Janus membrane) for the interfacial catalysis of lipase. Hydrophilic PS4VP was formed inside the macropores of PVDF facing the aqueous phase, while the pristine PVDF layer faced the organic phase. On account of the chemically functionalized PS channel walls, and the size being coordinated with the enzymes, CRL would be well absorbed in the pores of the PS4VP-filled PVDF layer. The self-assembly of PS4VP into well-ordered pores, with high porosity and low tortuosity, enhanced the transport of both substrates and products through the membrane at the interface. The immobilized lipase exhibited a three-fold higher specific activity than the free lipase, as well as the good reusability and long-term stability of the heterogeneous reaction. Briefly, membranes with nanoporosity, and immobilized lipase with a high catalysis efficiency and operation stability, were obtained via a simple approach. Thus, we expect that this work could not only make the Janus membrane useful in applications such as catalysis, biosensors and biomedical devices, but also provide an effective idea for the design and realization of nano-multiscale porous structures.

## Supplementary Materials

The following are available online at https://www.mdpi.com/2077-0375/10/8/162/s1, **Figure S1:** Two different structures of PS4VP-PVDF composite membranes. (a) PS4VP covered PVDF as a dual-layer membrane; (b) the PS4VP/PVDF Janus membrane, **Figure S2:** FT-IR spectrum of the surfaces of PS4VP/PVDF Janus membrane and PS4VP covered PVDF dual-layer membrane in comparison with pristine PVDF membrane, **Figure S3:** EDX image of the PS4VP-filled PVDF layer of the Janus membrane. Spectrum 1 is the PS4VP area, and spectrum 2 is the PVDF backbones, **Figure S4:** Enzyme loading process in the PS4VP/PVDF Janus membranes, **Figure S5:** BET adsorption curve of the Janus membrane before and after loading of CRL. (a) Original curve. (b) Derived pore size distribution curve calculated by BJH (Barret-Joyner-Halenda) method, **Figure S6:** TEM images of the PS4VP-filled PVDF layer of the Janus membrane. (a) Before CRL loading. (b) After CRL loading, **Figure S7:** Working curve the concentration and absorbance of the dye, **Figure S8:** Stress-strain curves of PVDF and PS4VP pristine membranes and PS4VP/PVDF Janus membrane. The inset shows the enlargement at small stress, **Table S1:** Hansen solubility parameters of relative polymers and solvents, **Table S2:** Mechanical properties calculated from stress-strain curves.
