# Supplementary material for: Amphipathic Janus Membrane with Hierarchical Multiscale Hyperporous Structure for Interfacial Catalysis
Source: Membranes (Basel). 2020 Jul 23;10(8):162. doi: 10.3390/membranes10080162 (PMC7465116; doi:10.3390/membranes10080162)
Supplement: Supplementary file 1 [file membranes-10-00162-s001.pdf]

Supporting Information

Amphipathic Janus Membrane with Hierarchical Multiscale Hyperporous Structure for Interfacial Catalysis

Yakai Lin [1#], Yuanyuan Liu [2#], Yicheng Su [1], Lin Wang [1], Yuanhui Tang [3], Tianyin Liu [1], Liwei Ren [4], and Xiaolin Wang [1*]

[1] Beijing Key Laboratory of Membrane Materials and Engineering, Department of Chemical Engineering, Tsinghua University, Beijing 100084, China; yk_lin@tsinghua.edu.cn
[2] Aerospace Institute of Advanced Materials & Processing Technology, China Aerospace Science & Industry Corp., Beijing 100084; liuyy07@163.com
[3] College of Chemistry and Environmental Engineering, China University of Mining and Technology, Beijing 100083, China; tangyuanhui@126.com
[4] College of Biological and Pharmaceutical Sciences, China Three Gorges University, Yichang 443002, China; renliwei@ctgu.edu.cn
[*] Correspondence: xl-wang@tsinghua.edu.cn

Figure S1 Two different structures of PS4VP-PVDF composite membranes. a) PS4VP covered PVDF as a dual-layer membrane; b) the PS4VP/PVDF Janus membrane........2

Figure S2 FT-IR spectrum of the surfaces of PS4VP/PVDF Janus membrane and PS4VP covered PVDF dual-layer membrane in comparison with pristine PVDF membrane........3

Figure S3 EDX image of the PS4VP-filled PVDF layer of the Janus membrane. Spectrum 1 is the PS4VP area, and spectrum 2 is the PVDF backbones........4

Table S1 Hansen solubility parameters of relative polymers and solvents........5

Figure S4 Enzyme loading process in the PS4VP/PVDF Janus membranes........6

Figure S5 BET adsorption curve of the Janus membrane before and after loading of CRL. a) Original curve. b) Derived pore size distribution curve calculated by BJH (Barret-Joyner-Halenda) method........7

Figure S6 TEM images of the PS4VP-filled PVDF layer of the Janus membrane. a) Before CRL loading. b) After CRL loading........8

Figure S7 Working curve the concentration and absorbance of the dye........9

Figure S8 Stress-strain curves of PVDF and PS4VP pristine membranes and PS4VP/PVDF Janus membrane. The inset shows the enlargement at small stress........10

Table S2 Mechanical properties calculated from stress-strain curves........10

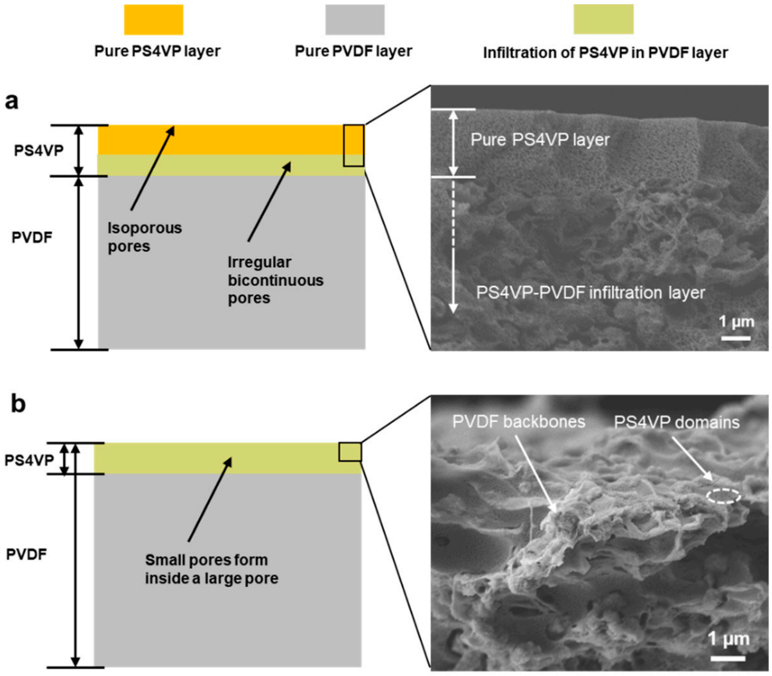

Figure S1 Two different structures of PS4VP-PVDF composite membranes. a) PS4VP covered PVDF as a dual-layer membrane; b) the PS4VP/PVDF Janus membrane

We fabricated two PS4VP-PVDF composite membranes. The PS4VP covered PVDF dual-layer membrane has been thoroughly reported with a dual-layer structure, in which PS4VP formed an independent layer on top of the surface of PVDF layer (Figure S1a). The PS4VP layer was fabricated through a SNIPS method. The thickness of the pure PS4VP layer is about 3 μm, and there is an infiltration layer beneath the interface of PS4VP pure layer and bulk PVDF. This structure is not benefit to heterogeneous interface catalysis. As the enzymes will be mainly retained in the pure PS4VP layer which immersed in water phase, on the one hand the reactants in oil phase have little chance to contact with the enzymes, on the other hand the extra thickness of the PS4VP layer will boost the transfer resistance while catalysis. To overcome this problem, a Janus membrane with a hierarchical multiscale hyperporous structure was fabricated with the PS4VP membrane filled in the macropores shaped by PVDF backbones (Figure S1b). On the membrane surface, the isoporous PS4VP regions were isolated by PVDF backbones. This kind of membrane had a much thinner nanoporous layer that favored molecular diffusion for heterogeneous catalysis.

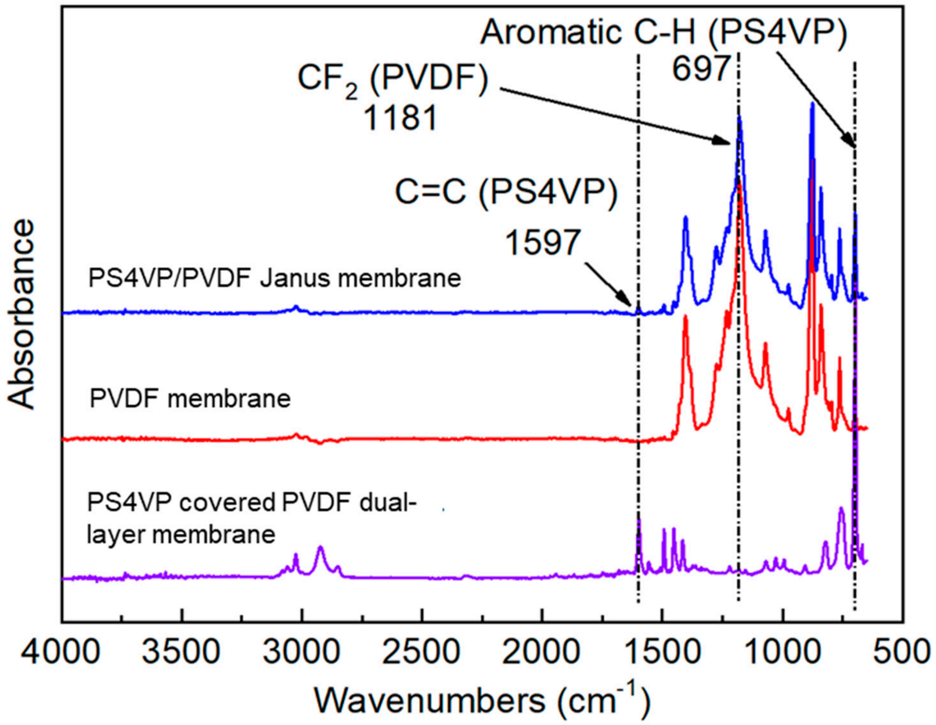

Figure S2 FT-IR spectrum of the surfaces of PS4VP/PVDF Janus membrane and PS4VP covered PVDF dual-layer membrane in comparison with pristine PVDF membrane.

FT-IR tests were proceeded to confirm the structures. Figure S2 shows the FT-IR spectrum of the surface of different membranes. For the PS4VP covered PVDF dual-layer membrane, the characteristic peaks at both 1597 cm-1 (C=C of PS4VP) and 697 cm-1 (aromatic C-H of PS4VP) indicated the existence of PS4VP. Furthermore, the missing characteristic peak at 1181 cm-1 (CF2 of PVDF) indicated that the PS4VP layer (Figure S1) entirely covered the beneath PVDF membrane. For the FTIR spectra of the PS4VP/PVDF Janus membrane, the characteristic peaks for both PVDF and PS4VP showed up, whereas the intensities of peaks ascribed to PS4VP were much weaker than that ascribed to PS4VP. This result was consistent with the observation (SEM figure in Figure S1b) that only a thin layer of PS4VP filled the PVDF macropores on the surface of the Janus membranes.

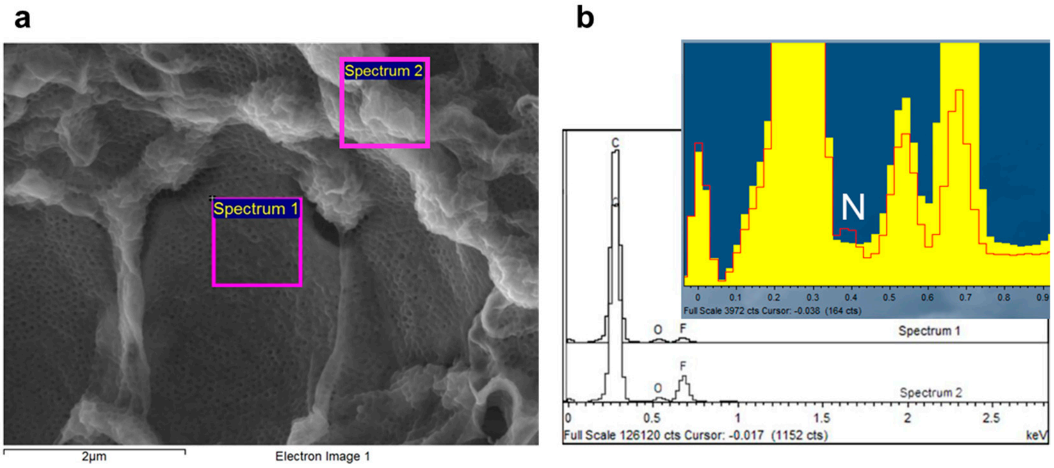

Figure S3 EDX image of the PS4VP-filled PVDF layer of the Janus membrane. Spectrum 1 is the PS4VP area, and spectrum 2 is the PVDF backbones.

The multiscale hyperporous structure can also be proved by EDX test. Surface SEM images shows that the well-ordered isoporous morphologies are divided by inrregular PVDF backbones. Two areas are chosen for test of EDX (Figure S3a). Spectrum 1 belongs to the area of PS4VP, while spectrum 2 belongs to the backbones of PVDF. Fluorine content in spectrum 1 is much lower than that of 2. This can prove the filling structure of PS4VP in PVDF pores. Because that the PS4VP only holds a single thin layer and PVDF is still in majority under beneath, fluorine can still be tested in spectrum 1, and nitrogen peak is relatively weak.

Table S1 Hansen solubility parameters of relative polymers and solvents.

**Polymer/ solvent**	$\delta_d(MPa^{1/2})$	$\delta_p(MPa^{1/2})$	$\delta_h(MPa^{1/2})$	$\delta_t(MPa^{1/2})$
**PS**	17.0	12.1	10.2	23.2
**Pyridinea**	19.0	8.8	5.9	21.8
**Dope solution**	17.7	7.3	8.4	20.9
**DMSO**	18.4	16.4	10.2	26.7
**TEP**	16.7	11.4	9.2	22.2
**DOX**	19.0	1.8	7.4	20.5
**TBP**	16.3	6.3	4.3	18.0

a. The parameter of P4VP blocks is replaced by pyridine.

The solubility of solvents can be characterized by Hansen solubility parameters. By listing the Hansen solubility parameters of the block copolymer and the ITAs (Table S1), it is found that the DOX has the nearest parameters compared with the original dope solution. δ_t stands for the total Hansen solubility parameter. δ_d, δ_p and δ_h stand for the dispersion, polarity and hydrogen parameter, respectively. The parameter of the dope solution (DMF: DOX: ACE=12:21:8 wt %) is just between that of PS blocks and pyridines. Nunes(ref) group suppose that the PS blocks contributes to the core while P4VP contributes to the shell when self-assembly in the dope solution. Increasing of the P4VP blocks will result in the morphology changes from hexagonal columnar to sphere shape. While using TBP as ITA, solvent exchange of dope solution and water will result in the decrease of δ_t. Finally the morphology exhibited the sphere shape.

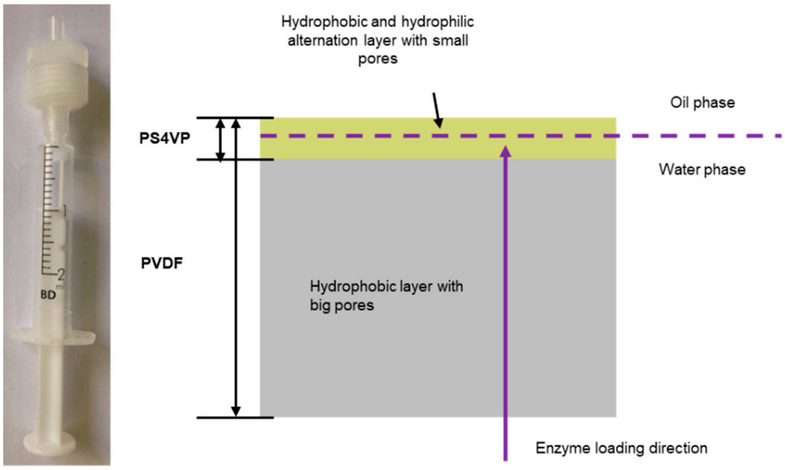

Figure S4 Enzyme loading process in the PS4VP/PVDF Janus membranes.

CRL loading process was executed with a syringe (Figure S4). Enzyme solution was slowly pushed through the Janus membranes. Finally, enzymes were retained in hydrophilic PS4VP layer, and luckily the interface of organic and aqueous phases is the best position for lipase to catalysis the reaction. If it is proceeded in the PS4VP covered dual-layer membrane, most of the enzymes cannot contact with the reactants in organic phase because of the very distinct interface of PVDF and PS4VP layers. Thus, the the PS4VP/PVDF Janus membranes are more fit for heterogeneous catalysis.

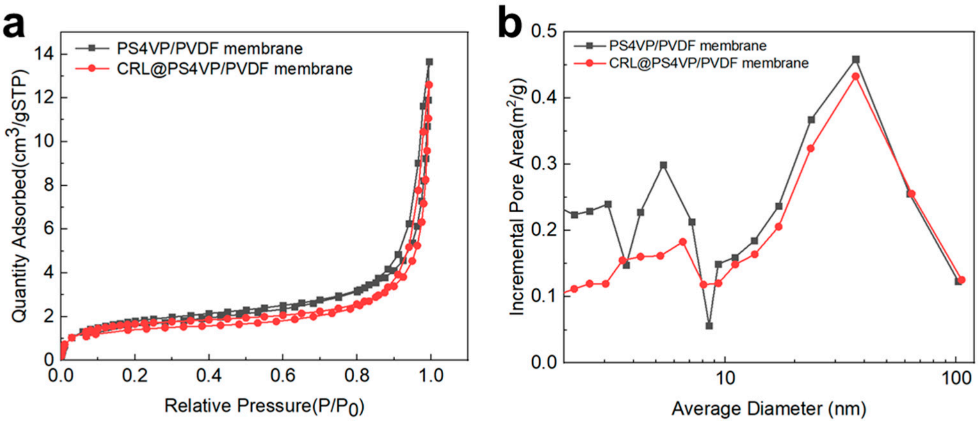

Figure S5 BET adsorption curve of the Janus membrane before and after loading of CRL. a) Original curve. b) Derived pore size distribution curve calculated by BJH (Barret-Joyner-Halenda) method.

BET curves of the Janus membrane changed before and after lipase loading (Figure S5). After absorbing of CRL, there is a dramatically decrease in the range of 2-10 nm, which indicates that CRL inside the PS4VP pore channels partially occupied the original adsorption volume. This helps prove the existence of CRL molecules in rather small PS4VP pore channels. The PS4VP layer consists of two parts with different pore sizes. On the top of several hundreds of nanometer range there are surface pores, and some of them are below 10 nm. There are still larger pores existed beneath the surface pores and they are at 20-100 nm range. After adsorption of nitrogen, the peek shows up at about 40 nm. However it is hard to tell whether CRL has been adsorbed in this area from BET curve as CRL is still too small compared with pore channels.

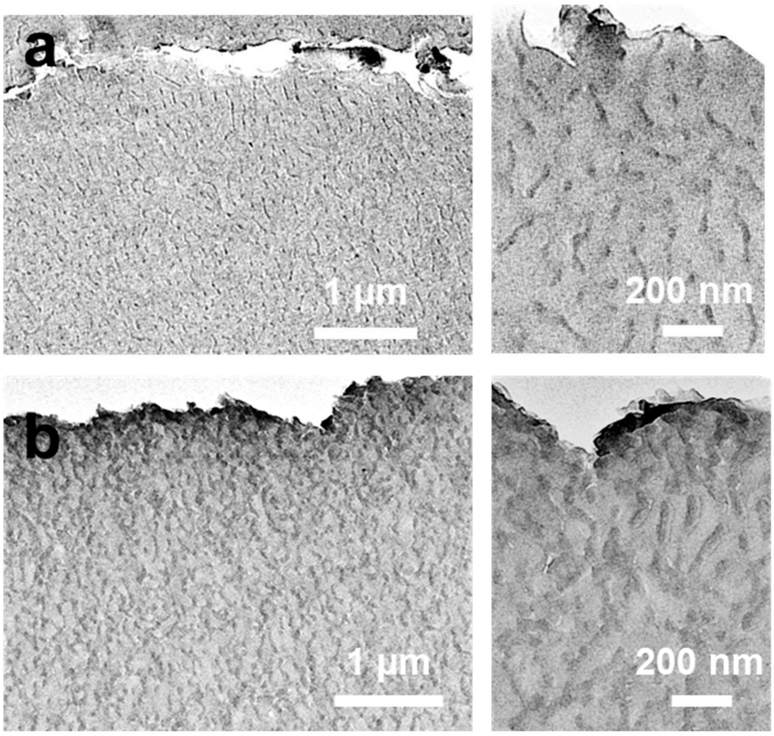

Figure S6 TEM images of the PS4VP-filled PVDF layer of the Janus membrane. a) Before CRL loading. b) After CRL loading.

The CRL absorbed in the pores of PS4VP-filled PVDF layer of the Janus membrane can be observed in TEM images as well (Figure S6). Comparing with the layer before and after lipase loading, the pore walls become thicker in TEM images. This indicated that enzyme molecules were absorbed onto the pore walls. Enzyme molecules can also be found in the enlarge picture in main text (Figure 3C).

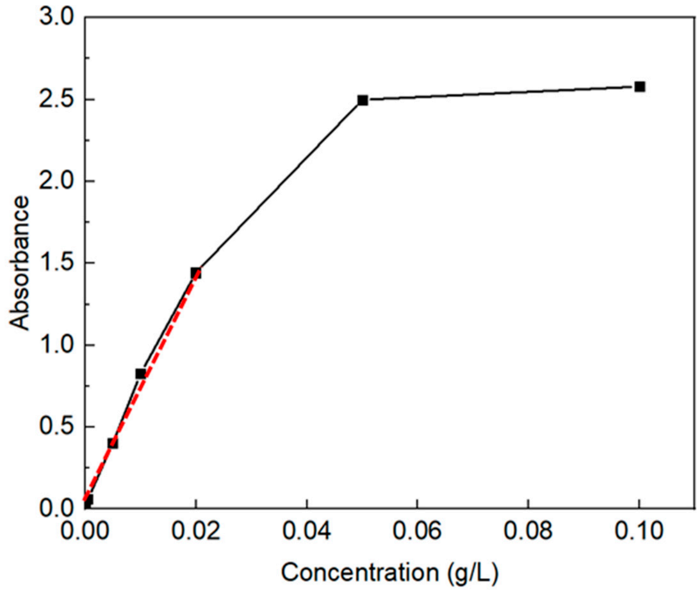

Figure S7 Working curve the concentration and absorbance of the dye.

In order to ensure the influence of enzyme loading on small molecules transport, the diffusion test employing a dye of 506 Da was proceeded. Figure S7 shows the working curve, as the test was measured by the UV spectrophotometer. The results shows freely pass of small molecules and proves that enzyme loading have little influence on the transfer of both substrates and products.

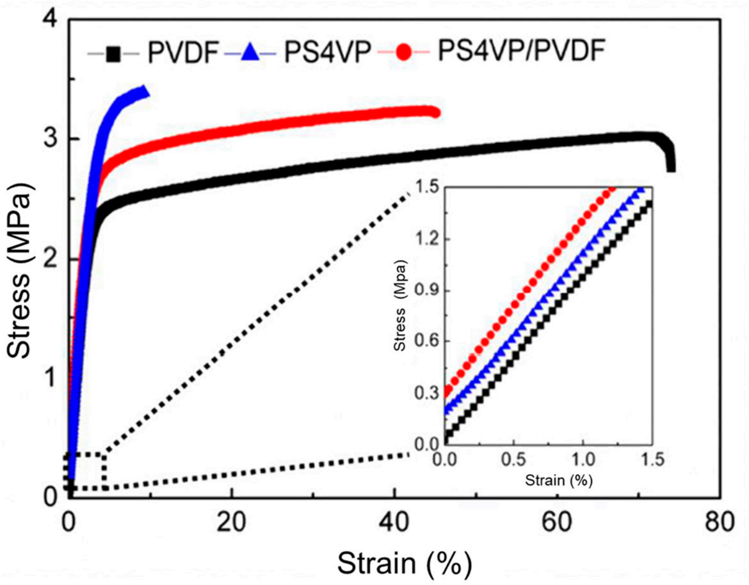

Figure S8 Stress-strain curves of PVDF and PS4VP pristine membranes and PS4VP/PVDF Janus membrane. The inset shows the enlargement at small stress.

Figure S8 illustrates the stress-strain curves of the PS4VP/PVDF Janus membrane in comparison to pristine PVDF and PS4VP membranes. The enhanced mechanical strength of Janus membrane (PS4VP/PVDF) indicated its superior robustness for long term use in contrast to the PS4VP membrane. Based on the stress-strain curves, the derived mechanical properties were calculated for further analysis (Table S2). The toughness is defined as the area enclosed by the stress-strain curve and the X axis. By combining the weak PS4VP and robust PVDF, the PS4VP-filled PVDF membrane showed both good elasticity and toughness. This is because that the critical stress on the weak PS4VP can be spread to robust PVDF in such composite structures, which enhance the compressive and tensile resistance.

Table S2 Mechanical properties calculated from stress-strain curves.

Sample	Elastic modulus	Tensile strength	Elongation at break (%)	Toughnes (MJ m^{-3})
**PVDF**	87.5	3.0	70.0	2.0
**PS4VP**	97.2	3.4	9.2	0.24
**PS4VP/PVDF**	92.5	3.2	45.0	1.3
